# Dietary protein and the glycemic index handle insulin resistance within a nutritional program for avoiding weight regain after energy-restricted induced weight loss

**DOI:** 10.1186/s12986-022-00707-y

**Published:** 2022-10-19

**Authors:** Fernando Vidal-Ostos, Omar Ramos-Lopez, Susan A. Jebb, Angeliki Papadaki, Andreas F. H. Pfeiffer, Teodora Handjieva-Darlenska, Marie Kunešová, Ellen E. Blaak, Arne Astrup, J. Alfredo Martinez, Diet, Obesity, and Genes (Diogenes) Project

**Affiliations:** 1 Escuela Internacional de Doctorado de la UNED, Bravo Murillo 39, Madrid, Spain; 2 Medicine and Psychology School, Autonomous University of Baja California, Universidad 14418, UABC, Parque Internacional Industrial Tijuana, 22390 Tijuana, B.C. Mexico; 3 Nuffield Department of Primary Care Health Sciences, University of Oxford, Oxford, UK; 4 Centre for Exercise, Nutrition and Health Sciences, School for Policy Studies, University of Bristol, Bristol, UK; 5 Department of Endocrinology, Diabetes and Nutrition, Charité Universitätsmedizin Berlin, German Center of Diabetes Research, DZD, Berlin, Germany; 6 Department of Pharmacology and Toxicology, Faculty of Medicine, Medical University, Sofia, Bulgaria; 7 Obesity Management Centre, Institute of Endocrinology, Prague, Czech Republic; 8 Department of Human Biology, NUTRIM, School for Nutrition and Translational Research in Metabolism, Maastricht University, Maastricht, The Netherlands; 9 Obesity and Nutrition Science, Novo Nordisk Fonden, Tuborg Havnevej 15, 2900 Hellerup, Denmark; 10 Navarra’s Health Research Institute (IdiSNA), Pamplona, Spain; 11 CIBERobn Physiopathology of Obesity and Nutrition, Carlos III Health Institute, Madrid, Spain; 12 Precision Nutrition Program, IMDEA Food Institute, CEI UAM + CSIC, Madrid, Spain

**Keywords:** Insulin resistance, TyG index, Protein diet, Glycemic index, Metabolic improvement, Precision nutrition

## Abstract

**Background and aim:**

The role of dietary protein and glycemic index on insulin resistance (based on TyG index) within a nutritional program for weight loss and weight maintenance was examined.

**Methods:**

This study analyzed 744 adults with overweight/obesity within the DIOGenes project. Patients who lost at least 8% of their initial weight (0–8 weeks) after a low-calorie diet (LCD) were randomly assigned to one of five ad libitum diets designed for weight maintenance (8–34 weeks): high/low protein (HP/LP) and high/low glycemic index (HGI/LGI), plus a control. The complete nutritional program (0–34 weeks) included both LCD plus the randomized diets intervention. The TyG index was tested as marker of body mass composition and insulin resistance.

**Results:**

In comparison with the LP/HGI diet, the HP/LGI diet induced a greater BMI loss (*p* < 0.05). ∆TyG was positively associated with resistance to BMI loss (β = 0.343, *p* = 0.042) during the weight maintenance stage. In patients who followed the HP/LGI diet, TyG (after LCD) correlated with greater BMI loss in the 8–34 weeks period (r = −0.256; *p* < 0.05) and during the 0–34 weeks intervention (r = −0.222, *p* < 0.05) periods. ΔTyG_1_ value was associated with ΔBMI_2_ (β = 0.932; *p* = 0.045) concerning the HP/LGI diet.

**Conclusions:**

A HP/LGI diet is beneficial not only for weight maintenance after a LCD, but is also related to IR amelioration as assessed by TyG index changes. Registration Clinical Trials NCT00390637.

**Supplementary Information:**

The online version contains supplementary material available at 10.1186/s12986-022-00707-y.

## Introduction

The World Health Organization defines obesity as a morbid accumulation of body fat often endangering health, which affects more than one billion people worldwide [1]. Hypertrophied and dysfunctional adipose tissue predisposes to the onset and progression of dyslipidemia, type 2 diabetes (T2D), and cardiovascular disease (CVD) [1]. Globally, CVD accounted for more than 50% of deaths in 2019, and is considered the leading cause of disability-adjusted life years around the world [2]. The etiology of CVD is multicausal, involving several risk factors including smoking, age, or unhealthy lifestyles, and morbid manifestations such as arterial hypertension, atherosclerosis, and overweight, among others [1, 3]. Indeed, obesity-associated insulin resistance (IR) is closely related to incidence of T2D and adverse cardiovascular events [1, 4].

In this context, the Triglyceride-glucose (TyG) index was identified as a valuable surrogate of IR [5–10]. A number of studies have confirmed the clinical utility of this marker as a good proxy for arterial hypertension [11], T2D [12], and non-alcoholic fatty liver disease [13]. Moreover, the TyG index is a reliable predictor for the development of obesity and CVD in different populations [6, 14], as well as T2D based on the combination of BMI and TyG index [15].

Concerning the available therapeutic strategies for the management of obesity, T2D and CVD, energy restriction induced weight loss and physical activity have been recommended as main practical approaches [1]. Indeed, weight loss is generally accompanied by multiple cardiometabolic benefits, including increased insulin sensitivity and improvements in circulating lipid profiles, blood pressure, and inflammation markers [16, 17]. Noteworthy, genetic, phenotypic, and environmental factors may contribute to the inter-individual differences in response to healthy lifestyle prescriptions [18], opening the door for personalized nutrition/medicine strategies for obesity or diabetes care and CVD prevention based on patient’s individualization through appropriate markers [19, 20].

Pharmacological and bariatric surgery treatments have been prescribed to patients with obesity alone or as co-adjuvants to nutritional advice under specific conditions [1, 3] Interestingly, personalized analytical markers related to adiposity are beginning to be implemented in order to provide personalized metabolic assistance to each patient [19]. Moreover, the macronutrient distribution of the diet may play a role in weight loss and maintenance [20, 21], within energy restricted or ad libitum diets, as in the Look AHEAD trial [22]. Certainly, nutritional interventions with different contents of fat, as in NUGENOB [23], protein, as in POUNDS LOST [24], have been investigated, in addition to the role of the glycemic index, as in DIOGenes [25], protein quality [26], or the Omega-3/Omega-6 fatty acid ratio [27]. A recent meta-analysis of randomized clinical trials revealed that a high dietary protein intake may induce specific beneficial cardiometabolic effects as compared to low-protein diets with potential impact on diabetes risk [28].

Currently, it is considered important to not only treat excessive body weight, but also combat individual morbid complications commonly associated with obesity (such as IR, hypertension or hypercholesterolemia), which require individualized approaches for diagnosis, long-term prognosis and treatments for precision medicine applications [29]. The aim of this study was to analyze the role of protein and glycemic index on IR based on the TyG index (a surrogate marker for IR) in patients with obesity within an integrative nutritional program designed to induce rapid weight loss and long-term body weight maintenance. A recent research (N = 19,420) reveled that elevated TyG index levels reflected a more severe IR and was associated with mortality due to all-cause and cardiovascular disease in a non-linear manner [30]. This research will facilitate the prediction of clinical outcomes in relation to insulin improvement, after dietary interventions based on low-calorie diets and following an adequate macronutrient distribution intake for avoiding weight regain.

## Material and methods

### Experimental design and cohort

The participants recruited in the current ancillary study belonged to the Diet, Obesity and Genes (DIOGenes) trial, which is a pan-European, multicenter, randomized controlled dietary intervention study [25, 31]. The aim of the DIOGenes project was to analyze the effect of protein (high/low intakes) and glycemic index (high/low) on weight maintenance after a weight loss of at least 8%, induced by a low-calorie diet (LCD) in overweight adults, as well as to describe the benefits on cardiometabolic risk factors associated with weight control [25]. The reference centers involved in the DIOGenes project were located in Denmark, Germany, the Netherlands, the United Kingdom, Greece (Crete), Bulgaria, Spain and the Czech Republic.

**Fig. 1:**
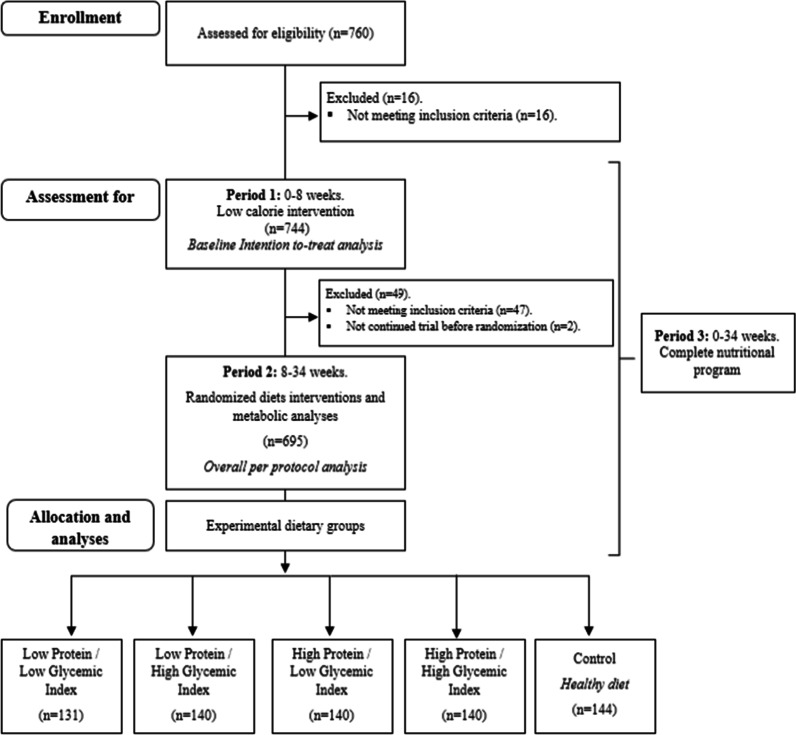
Flow chart and design concerning the participants enrolled in the current nutritional intervention

The participants in this study (n = 744) were enrolled between January 2006 and August 2007, comprising 259 males and 485 females, aged between 18–65 years and with BMI between 27–45 kg/m^2^. A diagram concerning the flow of patients included in the present analysis is depicted, including some details of the design and the label of the analyzed periods (Flowchart in Fig. 1). Some differences in the number of participants studied in the present study were attributed to the per protocol analysis or compared to the ITT analyses or the lack of some specific data concerning some variables. The exclusion criteria were as follows: Body weight changes higher than ± 3 kg within the last 2 months; pregnant or lactating women; subjects with heart, kidney, liver, psychiatric, endocrine and systemic infectious diseases; a history of gut malabsorption; hypertensive and/or hypercholesterolemic individuals with medication changes within the last 3 months; systolic blood pressure (SBP) > 160 and/or diastolic blood pressure (DBP) > 100 mmHg; and subjects consuming special diets. The complete methodological design and standard operating procedures have been previously described in detail [25, 31]. This study was registered at ClinicalTrials.gov as NCT00390637.

The procedures applied in the DIOGenes trial were in agreement with the Declaration of Helsinki and were approved by the local ethic committees corresponding to each participating country. An informed written consent was obtained from each participant included in this study [25].

### Measurements

Anthropometric, biochemical, and clinical measurements were performed at baseline, post-LCD and after the complete nutritional intervention, as previously described [25, 31]. Dual-energy x-ray absorptiometry (DEXA) or bioelectrical impedance analysis (BIA) were used to estimate body composition (body fat and fat-free mass percentage) depending on the participating study center. DEXA is an advanced technique for estimating body fat and lean soft tissue, which relies on the attenuation of radiation beams passing through the body to measure surface density [32]. After an overnight fast, participants assumed a stationary and supine position on the scanning bed with both arms pronated by their side to ensure reproducible positioning. Similarly, in the case of BIA, a weak electric current flows through the body and the voltage is measured in order to calculate impedance (resistance) of the body [33]. The analyses were adjusted considering the study center, which normalize body composition differences attributable to both analytical procedures. As usual, Body Mass Index (BMI) was calculated as weight (kg)/height (m^2^), while waist circumference was measured taking as reference the upper part of the hip bone and the lower part of the ribs [25]. Because % fat changes are not fully independently associated to body weight changes, which are influenced by the body size/height [34] or age [35], the fat free mass (FFM) index was calculated as follows: (FFM index = [(weight (kg) − fat (kg))/height^2^ (m)]) as described elsewhere [36]. Systolic blood pressure (SBP) and diastolic blood pressure (DBP) were evaluated following the criteria reported by the WHO [25, 31]. Mean arterial pressure (MAP) was calculated as (SBP + (2*DBP))/3, and pulse arterial pressure (PAP) as (SBP-DBP). Blood analytical markers such as glucose, triglycerides, total cholesterol, high-density lipoprotein cholesterol (HDL-c), c-reactive protein (CRP), creatinine, and fibrinogen were analyzed centrally according to standardized protocols [25, 31]. Low-density lipoprotein cholesterol (LDL-c) was calculated as total cholesterol (mg/dL)—HDL-c (mg/dL)—triglycerides (mg/dL)/5 [25]. The indices for estimating insulin resistance and pancreatic cell B functionality were calculated as: HOMA-IR = fasting insulin concentration (mIU/L) x fasting blood glucose concentration (mmol/L)/ 22.5; HOMA-B(%) = 20 × fasting insulin concentration (mIU/L)/fasting blood glucose concentration (mmol/L) and QUICKI index = 1/[log fasting plasma insulin (uU/ml) + log fasting blood glucose(mg/dl)], respectively [37]. The TyG index was calculated as Ln [TG (mg/dL)*glucose (mg/dL)/2] [5]. The TyG index was used since it is a simple, reliable, and inexpensive surrogate of insulin resistance as compared to measurements involving insulin [5–7]. Thus, higher TyG index is associated with more insulin resistance.

### Nutritional intervention

The DIOGenes trial was based on a nutritional intervention subsequently applied into two periods. In the first one (period 1), participants who met the inclusion criteria were prescribed a low-calorie diet (LCD) with a daily supply of 800 kcal during 8 weeks. Only individuals who lost at least 8% of their baseline weight were included in the second stage (period 2) for 26 weeks, and were randomly assigned to one of five weight maintenance intervention diets under a two-by-two factorial design: high protein (HP, 25% of total energy intake)/high glycemic index (HGI); HP/low glycemic index (LGI); low protein (LP, 13% of total energy intake)/HGI; and LP/LGI (Fig. 1). In addition, a control diet was used following the nutritional guidelines adapted to the country of each participating center, with a moderate protein content, and without restrictions regarding the glycemic index. The objective was to achieve a difference between HGI and LGI diets of at least 15 glycemic index (GI) units, as published [31]. All five diets were composed with a moderate fat content (25–30% of total energy intake), and unrestricted energy intake in order to assess the potential of diets to regulate appetite and body weight, as previously reported [25, 31]. The complete nutritional program comprised a total of 34 weeks (period 3), as described elsewhere [25, 31]. The present analysis focused on both 2 and 3 periods. The estimated energy deficit intake (EEDI) was calculated as reported elsewhere [38], and the result was converted from kcal to KJ using 4.18 as conversion factor. The glycaemic level of the diet will be determined on the basis of international GI tables [10]. The high and low GI diets will be designed to differ by 15 points on the GI scale [31], more information can be found at www.glycemicindex.com. Estimation of energy and nutrient intake were described elsewhere as well as glycemic index calculations [25, 31].

### Statistical analyses

In order to characterize the features of participants at baseline, descriptive statistics, based on *intention to treat* (ITT) analyses were calculated including means ± standard deviations (SD) for continuous variables and number (percentages) for categorical variables.

One-way analysis of variance (ANOVA) and the chi-square test (for continuous and categorical variables, respectively), were used to evaluate the differences between the randomized dietary groups at each step of the nutritional intervention, given the normality of the assessed variables. For differences in anthropometric and biochemical changes at period 2 (randomized diets considering from 8th week until 34th week) and period 3 (complete nutritional intervention considering; 0–34 weeks) that were previously statistically significant for ANOVA, Tukey's test was used for simultaneous statistical comparison between each type of diet. Participants with missing or negative values of ∆Weight_1_ (kg) and ∆Fat_1_ (%) after the LCD intervention (period 1) were excluded from the “*per protocol*” analyses, which involved only those volunteers whose phenotypical and analytical data were complete. The results of the *per protocol* analysis were preferred because better reflect the effects of the intervention when taken optimally, decreasing the probability of incurring a type II error, as described elsewhere [39].

Spearman's correlation tests were run to test the association of the TyG index at each period of the nutritional intervention with the subsequently dependent variables used in the linear regression models: ∆BMI_2_ (kg/m^2^) concerning the weight maintenance nutritional intervention (period 2; 8–34 weeks) and ∆BMI_3_ (kg/m^2^) concerning the complete nutritional program (period 3; 0–34 weeks).

Additional analyses were performed based on linear regression models following a “*per protocol*” approach. On the one hand, we used ∆BMI_2_ (kg/m^2^) the difference between final values of period 1 and period 2. On the other hand, ∆BMI_3_ (kg/m^2^) differences between the beginning and end of the total nutritional program were assessed. The baseline TyG index (TyG_1_), TyG index after the end of the intervention with the LCD (TyG_2_), as well as the TyG differences between both time points (∆TyG_1_), were used as predictor variables in the models.

Three regression models were fitted as follows: 1) a crude model (adjusted for the randomized diet (control healthy diet, LP/LGI, LP/HGI, HP/LGI, and HP/HGI; 2) a model adjusted for potential confounding variables such as sex, age, study center and randomized diet corresponding to the minimum setting model and; 3) maximum setting model was additionally adjusted for ∆Weight_1_ (kg), smoking status (nonsmoker, smoker, former smoker), daily walking time (< 15 min, 15–30 min, > 30 min), and alcohol intake (abstemious, throughout the week, at the weekend). For the estimation of the relationship between ΔTyG_1_ and BMI change during period 2; (8–34 weeks), independent multiple linear regression models were separately performed for each type of diet, based on the maximum fitted model (previously mentioned). Caloric intake was not used as a covariate to avoid colineality with weight loss in the maximum setting models. The reason is that we adjusted by body size, which is a surrogate of energy intake [40].

Statistical and graphical analyses were carried out with STATA 15 SE (StataCorp, College Station, TX). Statistical tests with an associated *p*-value lower than 0.05 were considered statistically significant, but some trends (*p* < 0.10) were also mentioned. Relevant specific results were also graphically illustrated.

## Results

The baseline clinical and metabolic characteristics of the total population distributed by the type of randomized diet are reported in Table 1. The mean BMI was 34.4 ± 4.6 kg/m^2^, weight 100.1 ± 17.7 kg, and body fat percentage 39.5 ± 7.9% (data not shown). The remaining variables concerning biochemical (glucose, triglycerides, total cholesterol, LDL-c, HDL-c, CRP, creatinine and fibrinogen), anthropometric/clinical (weight, height, waist, SBP, DBP, MAP and PAP) and lifestyle markers (smoking statues, daily walking time and alcohol intake) revealed the expected trends for a population with overweight/obesity (Table 1). Baseline HOMA-IR values among nutritional interventions (*p* > 0.05) were compatible with comparable are populations of subject with obesity, who also full-filled inclusion criteria. Furthermore, HOMA-B and QUICKI index values were compared among dietary groups, indicating that pancreatic functionality and insulin sensitivity, were similar at the beginning of the study, respectively. No significant differences concerning baseline variables by randomized diet categories were found (Table 1).

**Table 1:** Baseline clinical, metabolic and lifestyle characteristics of the enrolled population

N = 744	N	Control N = 151	LP/LGI N = 143	LP/HGI N = 149	HP/LGI N = 153	HP/HGI N = 146	*p*
Baseline variables		Mean ± SD	Mean ± SD	Mean ± SD	Mean ± SD	Mean ± SD	
Age (years)	742	42 ± 7	41 ± 6	41 ± 6	42 ± 7	42 ± 6	0.281
Sex	742	151	143	149	153	146	0.884
Male	258	52 (34.4%)	48 (33.6%)	49 (32.9%)	53 (34.6%)	52 (35.6%)	
Female	484	99 (65.6%)	95 (66.4%)	100 (67.1%)	100 (65.4%)	99 (67.8%)	
Weight (kg)	742	99.76 ± 17.49	100.41 ± 17.28	99.38 ± 16.88	99.51 ± 17.67	100.16 ± 18.47	0.986
*BMI (kg/m^2^)	742	34.43 ± 4.77	34.56 ± 5.52	34.48 ± 4.95	34.33 ± 4.74	34.10 ± 4.72	0.946
Waist (cm)	732	107.75 ± 12.75	107.79 ± 13.15	107.52 ± 13.03	106.87 ± 12.43	107.80 ± 13.54	0.963
Body fat (%)	651	40.60 ± 8.73	40.05 ± 9.65	40.21 ± 8.38	39.95 ± 9.87	39.56 ± 8.77	0.922
*FFM index	653	20.29 ± 2.30	20.44 ± 3.58	20.49 ± 2.62	20.39 ± 2.95	20.43 ± 2.26	0.984
Cholesterol (mg/dL)	742	190.45 ± 39.40	188.25 ± 43.60	189.45 ± 37.00	191.06 ± 37.80	190.24 ± 40.90	0.979
*LDL-c (mg/dL)	742	118.35 ± 35.10	118.43 ± 37.00	117.99 ± 31.30	118.40 ± 32.60	120.53 ± 35.30	0.972
*HDL-c (mg/dL)	742	47.59 ± 12.90	47.22 ± 12.50	46.88 ± 12.40	48.25 ± 14.00	45.74 ± 11.10	0.518
Triglycerides (mg/dL)	744	124.73 ± 62.90	115.04 ± 55.80	125.12 ± 62.60	124.21 ± 68.00	121.91 ± 52.30	0.597
Glucose (mg/dL)	736	90.66 ± 10.30	89.29 ± 11.80	88.37 ± 12.30	88.49 ± 12.60	89.05 ± 12.20	0.464
Insulin µIU/mL	712	11.93 ± 12.08	13.27 ± 15.13	10.63 ± 6.00	10.27 ± 5.97	12.09 ± 11.64	0.127
TyG index	736	8.51 ± 0.53	8.43 ± 0.49	8.50 ± 0.47	8.47 ± 0.57	8.50 ± 0.46	0.641
HOMA-IR	697	2.79 ± 2.91	3.20 ± 3.79	2.39 ± 1.43	2.41 ± 1.58	2.81 ± 2.55	0.055
HOMA-β (%)	697	144 ± 280.2	153.1 ± 188.8	151.7 ± 140.5	132.4 ± 90.8	179,.0 ± 243.7	0.399
QUICKI index	692	0.43 ± 0.23	0.4 ± 0.19	0.41 ± 0.12	0.43 ± 0.19	0.45 ± 0.43	0.573
U-C-peptide (nmol/24 h)	532	34.09 ± 40.53	34.97 ± 45.35	28.55 ± 27.48	28.96 ± 21.46	27.03 ± 21.77	0.260
*CRP (mg/L)	741	4.31 ± 4.73	5.22 ± 5.90	4.64 ± 5.08	4.80 ± 5.06	4.40 ± 5.24	0.593
Creatinine (mol)	710	8.86 ± 4.01	8.38 ± 4.36	8.20 ± 4.17	7.74 ± 3.76	8.33 ± 4.29	0.380
Fibrinogen (µmol/L)	735	9.37 ± 2.05	9.34 ± 1.94	9.43 ± 2.11	9.62 ± 2.54	9.12 ± 2.15	0.537
*SBP (mmHg)	641	127.89 ± 14.60	128.27 ± 15.40	126.48 ± 13.40	127.44 ± 13.90	129.81 ± 15.20	0.454
*DBP (mmHg)	641	81.06 ± 11.70	80.7 ± 11.70	80.74 ± 10.70	79.51 ± 11.40	81.67 ± 11.20	0.638
*MAP (mmHg)	641	96.67 ± 11.70	96.56 ± 11.60	95.99 ± 10.50	95.49 ± 11.10	97.72 ± 11.30	0.575
*PAP (mmHg)	641	46.84 ± 10.90	47.57 ± 12.60	45.74 ± 10.60	47.92 ± 11.20	48.13 ± 12.40	0.455
Smoking status	705	146 (100.0%)	134 (100.0%)	140 (100.0%)	145 (100.0%)	140 (100.0%)	0.129
Non-smoker		63 (43.2%)	56 (41.8%)	53 (37.9%)	67 (46.2%)	56 (40.0%)	
Former smoker		42 (28.8%)	52 (38.8%)	39 (27.9%)	45 (31.0%)	40 (28.6%)	
Smoker		41 (28.1%)	26 (19.4%)	48 (34.3%)	33 (22.8%)	44 (31.4%)	
Waking daily	689	137 (100.0%)	134 (100.0%)	134 (100.0%)	144 (100.0%)	140 (100.0%)	0.451
< 15 min		63 (46.0%)	52 (38.8%)	61 (45.5%)	65 (45.1%)	53 (37.9%)	
15–30 min		27 (19.7%)	41 (30.6%)	37 (27.6%)	39 (26.9%)	38 (27.1%)	
> 30 min		47 (34.3%)	41 (30.6%)	36 (26.9%)	40 (27.6%)	49 (35.0%)	
Alcohol weekly	737	148 (100.0%)	143 (100.0%)	149 (100.0%)	152 (100.0%)	145 (100.0%)	0.637
Abstemious		41 (27.2%)	60 (42.0%)	53 (35.6%)	39 (25.5%)	41 (28.1%)	
Throughout the week		40 (26.5%)	34 (23.8%)	35 (23.5%)	38 (24.8%)	37 (25.3%)	
At the weekend		67 (45.3%)	49 (34.3%)	61 (40.9%)	76 (50.0%)	67 (46.2%)	

Bold values indicate a *p* value < 0.05

Shown results are based on intention-to-treat analyses, and classified according of the type diet to which they were randomized post-LCD intervention

*Body mass index (BMI); fat free mass index (FFM I); low-density lipoprotein cholesterol (LDL-c); high-density lipoprotein cholesterol (HDL-c); C-reactive protein (CRP); systolic blood pressure (SBP); diastolic blood pressure (DBP); mean arterial pressure (MAP); pulse arterial pressure (PAP)

Diet types: Control (healthy diet), LP/LGI (low protein, low glycemic index diet), LP/HGI (low protein, high glycemic index diet), HP/LGI (high protein, low glycemic index diet), HP/HGI (high protein, high glycemic index diet)

Anthropometric and biochemical changes corresponding to period 3 (complete nutritional program) for each type of diet are reported in Table 2. Statistical comparisons tests showed differences in BMI (*p* = 0.037) between the LP/HGI and HP/LGI diets (Table 2). Additionally, for the multiple comparison tests, significant differences were found in HOMA (*p* = 0.013), insulin (*p* = 0.027) and fat (*p* = 0.037), concerning the differences between HP/HGI diet and LP/HGI diet (Table 2). Regardless of diet allocation, general reductions in waist, body fat percentage, triglycerides, CRP, and TyG index as well as blood pressure measurements were found (Table 2). Total cholesterol and HDL-c relatively increased in each of the diets, whereas LDL-c only increased in the HP/LGI diet (Table 2). Glucose levels were only reduced in the control and HP/LGI diets, although those changes were not statistically significant depending of each type of diet (Table 2).

**Table 2:** Anthropometric and biochemical changes at the complete nutritional program and the weight maintenance stage

†Diet Types	N	‡Period 2 (8–34 weeks)					*p*	N	‡Period 3 (0–34 weeks)					*p*
		Control	LP/LGI	LP/HGI	HP/LGI	HP/HGI			Control	LP/LGI	LP/HGI	HP/LGI	HP/HGI	
Variables		Effect size ± SD	Effect size ± SD	Effect size ± SD	Effect size ± SD	Effect size ± SD			Effect size ± SD	Effect size ± SD	Effect size ± SD	Effect size ± SD	Effect size ± SD	
*BMI (kg/m^2^)	489	0.16 ± 1.60 ^abcd^	−0.001 ± 1.70^abcd^	0.58 ± 1.56 ^abc^	−0.41 ± 2.21^abd^	−0.05 ± 1.68^abcd^	**0.004**	489	−3.78 ± 2.26 ^abcd^	−3.80 ± 2.35 ^abcd^	−3.22 ± 2.07 ^abc^	−4.27 ± 2.56 ^abd^	−3.97 ± 2.21 ^abcd^	**0.037**
Waist (cm)	479	0.72 ± 6.55	−0.001 ± 7.05	0.98 ± 7.75	−0.5 ± 7.20	−0.24 ± 6.72	0.546	482	−9.14 ± 7.21	−10.50 ± 7.99	−8.90 ± 8.40	−10.27 ± 7.33	−10.57 ± 7.28	0.402
Body fat (%)	406	−0.84 ± 3.73	−0.95 ± 4.73	−0.23 ± 3.99	−0.83 ± 4.80	−0.54 ± 7.19	0.909	410	−7.07 ± 5.08 ^abcd^	−7.01 ± 5.71 ^abcd^	−5.14 ± 3.74 ^abc^	−7.01 ± 4.88 ^abcd^	−7.69 ± 5.44 ^abd^	**0.037**
FFM Index	406	0.26 ± 1.06	0.19 ± 0.97	0.38 ± 0.90	−0.04 ± 1.31	0.12 ± 2.14	0.356	410	−0.21 ± 1.32	−0.06 ± 1.28	−0.43 ± 0.78	−0.49 ± 1.66	−0.02 ± 1.50	0.092
Cholesterol (mg/dL)	491	29.38 ± 30.10	27.18 ± 28.60	33.4 ± 35.80	30.16 ± 28.60	25.26 ± 26.60	0.418	491	0.25 ± 30.40	2.70 ± 35.30	2.40 ± 29.70	3.21 ± 32.60	1.43 ± 30.00	0.966
*LDL-c (mg / dL)	489	18.21 ± 25.10	15.13 ± 23.10	22.12 ± 30.00	18.87 ± 24.40	14.64 ± 23.80	0.263	490	−1.29 ± 25.80	−1.55 ± 27.30	−0.87 ± 25.30	0.77 ± 28.20	−2.41 ± 26.20	0.938
*HDL-c (mg / dL)	491	7.79 ± 9.00	9.36 ± 8.20	8.88 ± 10.30	8.24 ± 9.20	7.63 ± 8.20	0.634	491	4.85 ± 9.90	5.01 ± 11.50	5.76 ± 9.90	5.43 ± 7.70	5.94 ± 90.00	0.918
Triglycerides (mg/dL)	491	17.18 ± 42.10	16.43 ± 60.60	13.29 ± 55.00	15.36 ± 51.70	15.30 ± 35.60	0.988	491	−16.92 ± 51.20	−2.35 ± 63.20	−12.76 ± 48.50	−15.31 ± 51.60	−10.53 ± 47.70	0.336
Glucose (mg/dL)	479	1.77 ± 9.20	2.19 ± 10.50	4.15 ± 7.80	0.83 ± 11.00	0.96 ± 12.60	0.211	484	−2.19 ± 11.40	2.36 ± 13.30	0.51 ± 14.50	−0.02 ± 14.90	0.09 ± 12.60	0.214
Insulin (µUI/ML)	406	1.11 ± 3.70	1.12 ± 7.73	3.18 ± 9.30	1.53 ± 3.80	0.99 ± 5.06	0.174	432	−2.94 ± 6.91 ^abcd^	−2.26 ± 9.12 ^abcd^	−0.51 ± 9.44 ^abc^	−2.27 ± 5.32 ^abcd^	−5.12 ± 12.84 ^abc^	**0.027**
TyG index	478	0.18 ± 0.40	0.17 ± 0.49	0.20 ± 0.42	0.12 ± 0.43	0.12 ± 0.40	0.668	483	−0.14 ± 0.41	0.01 ± 0.48	−0.09 ± 0.42	−0.09 ± 0.49	−0.09 ± 0.43	0.221
HOMA-IR	398	0.29 ± 0.89	0.35 ± 2.43	0.81 ± 1.96	0.36 ± 1.09	0.27 ± 1.46	0.264	420	−0.73 ± 1.69 ^abcd^	−0.62 ± 2.70 ^abcd^	−0.06 ± 2.00 ^abc^	−0.57 ± 1.51 ^abcd^	−1.30 ± 2.78 ^abd^	**0.013**
C-Peptide (nmol/24 h)	-	−	−	−	−	−	−	297	−13.39 ± 39.9	−14.90 ± 39.22	−4.96 ± 16.64	−10.8 ± 19.52	−6.67 ± 16.80	0.2867
*CRP (mg/L)	488	−0.22 ± 7.68	−1.08 ± 5.66	0.22 ± 2.63	−0.77 ± 2.24	0.38 ± 4.46	0.298	489	−0.86 ± 3.06	−1.88 ± 4.95	−1.06 ± 4.36	−1.93 ± 2.77	−1.02 ± 5.08	0.182
*SBP (mmHg)	476	4.08 ± 12.80	5.07 ± 13.00	5.29 ± 10.90	3.86 ± 14.20	2.65 ± 13.60	0.668	404	−7.28 ± 11.90	−6.41 ± 11.30	−6.41 ± 11.50	−5.85 ± 12.90	−6.74 ± 11.80	0.955
*DBP (mmHg)	476	3.07 ± 8.40	1.36 ± 8.00	3.47 ± 7.50	1.66 ± 7.80	1.23 ± 80.00	0.184	404	−6.75 ± 8.70	−7.59 ± 8.50	−7.71 ± 9.70	−5.76 ± 9.20	−6.99 ± 8.60	0.632
*MAP (mmHg)	476	3.40 ± 8.70	2.59 ± 8.50	4.08 ± 7.70	2.39 ± 8.70	1.70 ± 9.10	0.385	404	−6.98 ± 8.40	−7.20 ± 7.60	−7.27 ± 9.20	−5.79 ± 9.40	−6.91 ± 8.20	0.791
*PAP (mmHg)	476	1.01 ± 10.90	3.71 ± 11.00	1.82 ± 9.10	2.20 ± 12.00	1.42 ± 9.70	0.473	404	−0.45 ± 11.30	−1.18 ± 12.20	−1.3 ± 10.00	−0.10 ± 10.30	−0.25 ± 11.10	0.815
*EEDI (KJ/week)	489	1.09 ± 4.43	0.79 ± 4.05	1.59 ± 3.76	−0.17 ± 5.48	0.5 ± 8.95	0.356	489	−0.88 ± 5.52	−0.25 ± 5.35	−1.8 ± 3.26	−2.05 ± 6.94	−0.08 ± 6.27	0.092

Bold values indicate a *p* value < 0.05

Shown results are based on *per-protocol* analyses

^†^Types of diets: Control (healthy diet), LP/LGI (low protein, low glycemic index diet), LP/HGI (low protein, high glycemic index diet), HP/LGI (high protein, low glycemic index diet), HP/HGI (high protein, high glycemic index diet)

*Body mass index (BMI); fat free mass index (FFM I); low-density lipoprotein cholesterol (LDL-c); high-density lipoprotein cholesterol (HDL-c); estimated energy deficit intake (EEDI); C-reactive protein (CRP); systolic blood pressure (SBP); diastolic blood pressure (DBP); mean arterial pressure (MAP); pulse arterial pressure (PAP)

^‡^Period 2: Corresponds to the differences between the parameters after the low-calorie diet intervention and after the nutritional treatment focused on weight maintenance for each type of randomized diet (8–34 weeks); Period 3: Corresponds to the differences between baseline and final parameters encompassing the complete nutritional period (during 34 weeks)

Superindex (abcd): Differences with the same letter are not significantly different from each other at the 5% significance level

The changes concerning the weight maintenance stage with the five types of randomized diets (period 2) are also shown in Table 2. In the maintenance stage there were no differential decreases (*p* > 0.05) in body fat percentage and CRP or in total cholesterol, HDL-c, Triglyceride, glucose, TyG index, SBP, DBP, MAP and PAP among dietary groups, despite some assumed beneficial trends, were found (Table 2).

The EEDI (period 2; 8–34 weeks) corresponds to the average energy deficit intake during the weight maintenance phase, depending on the type of diet selected, while EEDI 1–3 (period 3; 34 weeks) indicates the estimated energy deficit corresponding to the complete nutritional diet. The greater energy deficit intake was associated with more weight loss and better insulin sensitivity. Energy intake deficit ranged from −2470 kcal (10,324.6 kJ) to −2198 kcal (9187.64 kJ) among dietary groups, protein intake increased from 4.5 to 6.1% in the high protein groups while remained stable in the normal protein group (−0.1 to −0.2%), meanwhile glycemic index changes were (−4.3 to −4.7%) in the low glycemic index compared with 0 to 0.7% in the high glycemic index group [25].

Linear regression models following a *per protocol* approach to estimate BMI outcomes using TyG values as predictors are reported in Tables 3 and 4. In the total time (0–34 weeks) of the nutritional intervention (period 3), the maximum fitted model revealed a tendency (not statistical significant) to lower BMI loss per each unit of increase of the ∆TyG_1_ index (the higher ∆TyG_1_ index, the lower BMI loss), as reported in Table 3 (β = 0.339, *p* = 0.051). Patients assigned to the HP/LGI diet showed higher BMI losses in the maximum setting models (*p* < 0.05) compared to "healthy diet" as a proper control (Table 3).

**Table 3:** Regression models based on TyG to estimate BMI changes within on the complete nutritional program; period 3 (0–34 weeks)

Change in BMI (kg/m^2^) ‡Period 3 (0–34 weeks)	TyG	β (95% CI)	*p*	R^2^	TyG	β (95% CI)	*p*	R^2^	TyG	β (95% CI)	*p*	R^2^
*Crude*	TyG _1_	0.185 (−0.221; 0.590)	0.371	0.0228*	TyG _2_	−0.154 (−0.637; 0.330)	0.532	0.0219	∆TyG_1_	0.350 (−0.083; 0.783)	0.113	0.0267*
	LP/LGI	0.013 (−0.635; 0.661)	0.969		LP/LGI	0.002 (−0.653; 0.657)	0.996		LP/LGI	0.058 (−0.602; 0.717)	0.863	
	LP/HGI	0.560 (−0.100; 1.220)	0.096		LP/HGI	0.564 (−0.102; 1.229)	0.097		LP/HGI	0.572 (−0.095; 1.238)	0.092	
	HP/LGI	−0.504 (−1.127; 0.119)	0.113		HP/LGI	−0.497 (−1.127; 0.133)	0.122		HP/LGI	−0.497 (−1.128; 0.134)	0.122	
	HP/HGI	−0.161 (−0.805; 0.483)	0.624		HP/HGI	−0.138 (−0.787; 0.511)	0.676		HP/HGI	−0.090 (−0.744; 0.563)	0.786	
†*Minimum setting*	TyG _1_	0.179 (−0.239; 0.596)	0.400	0.0685***	TyG _2_	−0.056 (−0.536; 0.425)	0.820	0.0666**	∆TyG_1_	0.258 (−0.182; 0.699)	0.249	0.0686**
	LP/LGI	−0.016 (−0.651; 0.620)	0.961		LP/LGI	−0.047 (−0.690; 0.595)	0.885		LP/LGI	0.006 (−0.642; 0.654)	0.985	
	LP/HGI	0.590 (−0.058; 1.238)	0.074		LP/HGI	0.585 (−0.069; 1.239)	0.080		LP/HGI	0.597 (−0.058; 1.253)	0.074	
	HP/LGI	−0.531 (−1.143; 0.080)	0.088		HP/LGI	−0.531 (−1.150; 0.088)	0.092		HP/LGI	−0.518 (−1.139; 0.103)	0.102	
	HP/HGI	−0.230 (−0.863; 0.403)	0.476		HP/HGI	−0.225 (−0.863; 0.414)	0.489		HP/HGI	−0.178 (−0.822; 0.467)	0.588	
†*Maximum setting*	TyG_1_	0.118 (−0.212; 0.449)	0.483	0.4674***	TyG _2_	−0.243 (−0.612; 0.127)	0.197	0.4698***	∆TyG_1_	0.339 (−0.001; 0.679)	0.051	0.4733***
	LP/LGI	−0.260 (−0.760; 0.239)	0.306		LP/LGI	−0.291 (−0.795; 0.212)	0.256		LP/LGI	−0.246 (−0.754; 0.261)	0.341	
	LP/HGI	0.315 (−0.193; 0.823)	0.224		LP/HGI	0.332 (−0.181; 0.844)	0.204		LP/HGI	0.326 (−0.186; 0.837)	0.211	
	HP/LGI	−0.833 (−1.310; −0.356)	**0.001**		HP/LGI	−0.804 (−1.287; −0.321)	**0.001**		HP/LGI	−0.808 (−1.289; −0.326)	**0.001**	
	HP/HGI	−0.244 (−0.738; 0.249)	0.331		HP/HGI	−0.261 (−0.757; 0.235)	0.302		HP/HGI	−0.216 (−0.716; 0.283)	0.395	

Bold values indicate a *p* value < 0.05

Triglycerides-glucose index (TyG): TyG_1_ (basal value; pre-LCD), TyG_2_ (8 weeks; post-LCD) and the TyG changes for period 1 (ΔTyG_1_)

Regression coefficients (β); 95% confidence interval (95% CI) and R^2^

* R^2^ *p*-value * < 0.05; ** < 0.001; *** < 0.0001

Highlight: statistical association

^†^Minimum setting model included (sex, age (years), center, and randomized diet); maximum setting model included (sex, age (years), center, ∆weight_1-2_ (kg), smoking status (nonsmoker, smoker, former smoker); daily walking time (< 15 min, 15–30 min, > 30 min), alcohol intake (abstemious, throughout the week, at the weekend) and randomized diet

^‡^Period 3: Corresponds to the differences between baseline and final parameters encompassing the complete nutritional program (during 34 weeks)

Types of diets: LP/LGI (low protein, low glycemic index diet), LP/HGI (low protein, high glycemic index diet), HP/LGI (high protein, low glycemic index diet), HP/HGI (high protein, high glycemic index diet)

**Table 4:** Regression models based on TyG to estimate BMI changes within on the weight maintenance stage; period 2 (8–34 weeks)

Change in BMI (kg/m^2^) ‡Period 2 (8–34 weeks)	TyG	β (95% CI)	*p*	R^2^	TyG	β (95% CI)	*p*	R^2^	TyG	β (95% CI)	*p*	R^2^
*Crude*	TyG _1_	0.283 (−0.029; 0.595)	0.076	0.038*	TyG _2_	−0.245 (−0.617; 0.127)	0.196	0.035*	∆TyG_1_	0.515 (0.184; 0.846)	**0.002**	0.0504**
	LP/LGI	−0.136 (−0.635; 0.362)	0.591		LP/LGI	−0.142 (−0.646; 0.362)	0.581		LP/LGI	−0.078 (−0.583; 0.426)	0.761	
	LP/HGI	0.424 (−0.084; 0.932)	0.101		LP/HGI	0.432 (−0.080; 0.945)	0.098		LP/HGI	0.443 (−0.067; 0.953)	0.088	
	HP/LGI	−0.582 (−1.061; −0.102)	**0.018**		HP/LGI	−0.555 (−1.040; −0.070)	**0.025**		HP/LGI	−0.543 (−1.026; −0.060)	**0.028**	
	HP/HGI	−0.185 (−0.681; 0.311)	0.464		HP/HGI	−0.178 (−0.677; 0.322)	0.485		HP/HGI	−0.120 (−0.621; 0.380)	0.637	
†*Minimum setting*	TyG _1_	0.185 (−0.132; 0.502)	0.252	0.1074***	TyG _2_	−0.168 (−0.532; 0.196)	0.365	0.1074***	∆TyG_1_	0.351 (0.018; 0.684)	**0.039**	0.1136***
	LP/LGI	−0.183 (−0.666; 0.299)	0.456		LP/LGI	−0.195 (−0.682; 0.292)	0.431		LP/LGI	−0.151 (−0.641; 0.339)	0.545	
	LP/HGI	0.418 (−0.074; 0.909)	0.096		LP/HGI	0.424 (−0.072; 0.919)	0.094		LP/HGI	0.435 (−0.061; 0.930)	0.086	
	HP/LGI	−0.638 (−1.102; −0.173)	**0.007**		HP/LGI	−0.614 (−1.083; −0.145)	**0.010**		HP/LGI	−0.598 (−1.067; −0.128)	**0.013**	
	HP/HGI	−0.287 (−0.768; 0.194)	0.242		HP/HGI	−0.286 (−0.770; 0.197)	0.245		HP/HGI	−0.238 (−0.725; 0.249)	0.338	
† *Maximum setting*	TyG_1_	0.139 (−0.182; 0.459)	0.378	0.1864***	TyG _2_	−0.227 (−0.585; 0.131)	0.225	0.1901***	•∆TyG_1_	0.343 (0.013; 0.673)	**0.042**	0.1951***
	LP/LGI	−0.226 (−0.721; 0.248)	0.360		LP/LGI	−0.258 (−0.746; 0.230)	0.324		LP/LGI	−0.218 (−0.710; 0.274)	0.412	
	LP/HGI	0.320 (−0.178; 0.808)	0.203		LP/HGI	0.331 (−0.165; 0.828)	0.185		LP/HGI	0.326 (−0.170; 0.822)	0.191	
	HP/LGI	−0.780 (−1.257; −0.332)	**0.001**		HP/LGI	−0.757 (−1.225; −0.289)	**0.002**		HP/LGI	−0.759 (−1.226; −0.292)	**0.002**	
	HP/HGI	−0.216 (−0.703; 0.255)	0.375		HP/HGI	−0.232 (−0.713; 0.248)	0.364		HP/HGI	−0.190 (−0.674; 0.294)	0.464	

Bold values indicate a *p* value < 0.05

Triglycerides-glucose index (TyG): TyG_1_ (basal value; pre-LCD), TyG_2_ (8 weeks; post-LCD) and the TyG changes for period 1 (ΔTyG_1_)

Regression coefficients (β); 95% confidence interval (95% CI) and R^2^

*R^2^ *p*-value * < 0.05; ** < 0.001; *** < 0.0001

Highlight: statistical association

^†^Minimum setting model included (sex, age (years), center, and randomized diet); maximum setting model included (sex, age (years), center, ∆weight_1-2_ (kg), smoking status (nonsmoker, smoker, former smoker); daily walking time (< 15 min, 15–30 min, > 30 min), alcohol intake (abstemious, throughout the week, at the weekend) and randomized diet

^‡^Period 2: Corresponds to the differences between the parameters after the low-calorie diet intervention and after the nutritional treatment focused on weight maintenance for each type of randomized diet (34–8 weeks)

Types of diets: LP/LGI (low protein, low glycemic index diet), LP/HGI (low protein, high glycemic index diet), HP/LGI (high protein, low glycemic index diet), HP/HGI (high protein, high glycemic index diet)

• On regressions models, matching diet type with its corresponding ΔTyG_1_ value, only a significant β value: 0.932 (*p*-value: 0.045) concerning the HP/LGI diet was found

During the weight maintenance (period 2), the ∆TyG_1_ index was associated with lower BMI reductions (also, the higher ∆TyG_1_ index, the lower BMI decrease) in each of the models analyzed, specifically in the maximum setting model (β = 0.343, *p* = 0.042) as shown in Table 4. In all multiple linear regression models, participants consuming a HP/LGI diet lost more BMI compared to the rest of dietary groups (*p* < 0.05) and with the "healthy diet" as a proper control (Table 4). After performing separate multiple linear regressions (with appropriate adjustments), matching diet type with its corresponding ΔTyG_1_ value, we observed only one significant β value: 0.932 (*p*-value: 0.045), relative to the HP/LGI diet. While an association (β = 2.58; *p* ≤ 0.001; data no shown in tables) was found between ΔHOMA_2_ (8–34 weeks) and ΔTyG_2_ (8–34 weeks) concerning HP/LGI.

The baseline TyG_1_ positively correlated with changes in BMI concerning the period 2 (r = 0.3140, *p* < 0.05) following the HP/HGI diet and in period 3 (r = 0.2680, *p* < 0.001) (Additional file 1: Table S1). Meanwhile, the TyG_2_ (after LCD) correlated with a greater loss of BMI in period 2 (r = −0.2560, *p* < 0.05) in participants assigned to the HP/LGI diet and in period 3 (r = −0.2220, *p* < 0.05) as reported in Additional file 1: Table S1. The ∆TyG_1_ positively correlated with resistance to BMI loss (the higher TyG, the lower BMI loss) in both periods with the HP/HGI diet (Additional file 1: Table S1). Interestingly, the correlations of TyG with HOMA-IR are highly statistically significant both in the baseline of period 1 (r = 0.2364, *p* < 0.0001) and at the start of period 2 (r = 0.2453, *p* < 0.0001). Furthermore, no statistical differences (*p* > 0.05) were found concerning HOMA-IR values depending on the dietary experimental groups.

**Fig. 2:**
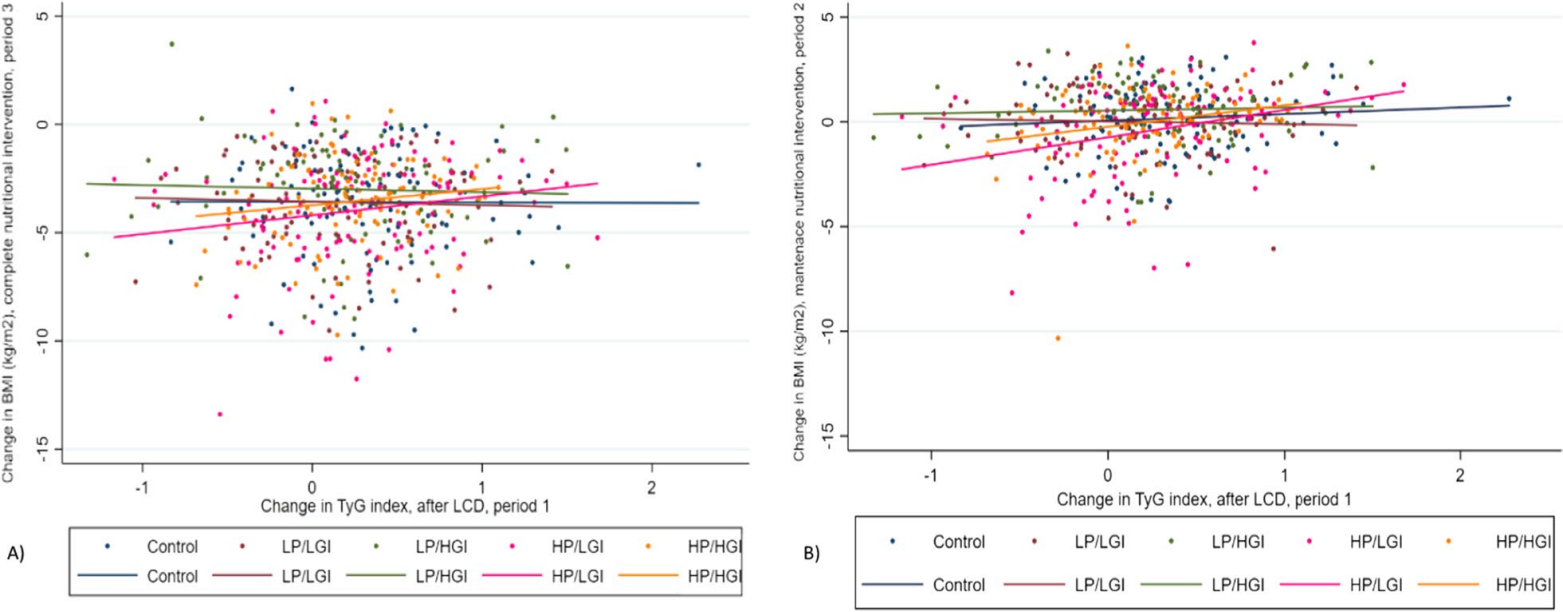
Change in TyG index after LCD and modifications in BMI (kg/m^2^) for each type of randomized diet, **A** Change in BMI (kg/m^2^) corresponding to the complete nutritional intervention, (period 3, 0–34 weeks); **B** Change in BMI (kg/m^2^) concerning the maintenance nutritional intervention, (period 2, 8–34 weeks). Results shown are based on ITT analysis. Types of diets: Control (healthy diet), LP/LGI (low protein, low glycemic index diet), LP/HGI (low protein, high glycemic index diet), HP/LGI (high protein, low glycemic index diet), HP/HGI (high protein, high glycemic index diet). Only significant differences were obtained between Low Protein/High Glycemic Index and High Protein/Low Glycemic Index (HP/LGI) diet (*p* = 0.015). Period 3: Corresponds to the differences between baseline and final parameters, encompassing the complete nutritional period (during 34 weeks); Period 2: Corresponds to the differences between the parameters after the low-calorie diet intervention (8 weeks) and after the nutritional treatment focused on weight maintenance for each type of randomized diet (8–34 weeks)

Correlations between TyG index values after LCD and modifications in BMI for each type of randomized diet are plotted (Fig. 2). Participants with higher levels of TyG (more insulin resistance) tended to reduce their BMI less (the higher TyG, the lower BMI loss) during in the total nutritional intervention (period 3) as illustrated (Fig. 2A), and also in the weight maintenance stage (period 2) as illustrated (Fig. 2B). In both intervention periods, a clear tendency to lose more BMI was found in participants who received the HP/LGI diet compared to the other dietary groups (Fig. 2).

## Discussion

A number of nutritional trials concerning obesity management and accompanying comorbidities, such as T2D and CVD, have mainly been focused on weight loss [1], whereas putative benefits related to specific pathophysiological mechanisms (including IR) are less frequently considered [1, 41]. Furthermore, although attention has been paid to the role of glycemic index and fiber in obesity and CVD management, less information is available regarding the effect of protein intake [42–44]. The present research focused on examining the concomitant effect of glycemic index and protein intake on insulin status within the DIOGenes trial [25, 45, 46]. In this context, the TyG index, a composite marker of fasting glucose and triacylglycerols, has shown to be a useful and reliable predictor of IR [5], T2D [12] and CVD [14, 47] in diverse populations.

The anthropometric/biochemical baseline data from the current ancillary study were in line with results reported by comparable studies such as The POUNDS LOST trial [24], LOOK AHEAD [22], ASKED [41], NUGENOB [23], and PREVIEW [17].

Interestingly, in the complete nutritional intervention (period 3) and the dietary randomization stage (period 2), participants treated with the HP/LGI diet reduced more the BMI, compared to those in the LP/HGI diet. Moreover, the HP/LGI diet correlated positively with better BMI maintenance (even further reduction of BMI) in comparison with the other types of diets, whereas the LP/HGI diet showed the worst BMI maintenance (regain). Current analyses provided a valuable evidence that a weight management program based on a LCD (8 weeks) followed by a specific dietary macronutrient distribution (8–34 weeks) within a strategy to avoid weight regain can be better achieved with an ad libitum HP/LGI dietary regime, where the initial LCD may have a determinant role. Considering the protein content of this particular diet (25% of total energy intake), this finding is in agreement with previous investigations reporting greater weight loss in patients following a high-protein diet compared to a low-protein diet [48, 49]. For instance, a high-protein intake showed weight loss benefits in the POUNDS LOST trial, although carbohydrates were not demonstrated to influence this improvement [24]. Some of these previous results concerning the weight loss in the maintenance period was greater in the HP/LGI group, with an average weight loss of around 0.5 kg, which may be attributable to the positive effects of high-protein diets in relation to diet-induced thermogenesis [44, 48, 50], greater satiety at mealtimes [44, 48, 51], and increased lean mass preservation [48], which could contribute to enable promote a negative energy balance, thus inducing weight loss and fat reduction [44]. Additionally, hypoenergetic diets induced weight loss decreases appetite perceptions and preference for high-fat/high-carbohydrate foods within the DIOGenes trial [52], which is of interest when interpreting current outcomes.

Also, dietary protein has been associated with improvements in glucose homeostasis and insulin sensitivity [44, 53], although this effect is well known to occur after the intake of high-fiber, low-glycemic index foods [24, 25]. Besides, participants with higher IR benefit more from a low-glycemic index diet apparently due to a lower insulin demand to metabolize the dietary carbohydrates in the circulatory stream [54, 55]. Additionally, diets with moderately high protein content and low glycemic index could modulate caloric intake [48]. Both dietary components have been associated with anti-inflammatory benefits [3, 46], as appreciated concerning decreases of CRP concentrations, which may be indirectly related to body fat reductions and improvement of insulin sensitivity [21, 56]. Together, these outcomes are earlier trials associating high-protein diets with successful weight loss and weight maintenance, as well as reduced cardiometabolic risk factors, including blood pressure, lipids, and inflammation [46, 48], associated to low glycemic index [55, 57]. Furthermore, some HOMA relationships with insulin resistance had been previously published [31, 58]. Indeed, an ancillary study, involving the Diogenes intervention analyzing insulin response reported that the insulin response was lower in the HP/LGI after 60 and 90 min of the OGTT at the end of the 6-months intervention (*p* < 0.05) as compared to the other dietary groups [58]. The source of protein may be important concerning insulin and HOMA-IR responses [31]. Interestingly, a statistical association between ΔHOMA_2_ and ΔTyG_2_ was found for the HP/LGI diet, concerning to period 2 (8–34 weeks), thus reinforcing the idea of the utility value of TyG as a proxy of insulin resistance, showing an improvement after following a diet moderately high in protein and consuming carbohydrates with a low glycemic index.

The baseline TyG (TyG_1_), TyG after LCD (TyG_2_) and the TyG differences between both time points (∆TyG_1_) had some predictive value regarding BMI loss even after adjustment for variables such as sex, age, center, ∆Weight_1_, smoking status, daily walking time, and alcohol intake. Of note, only ΔTyG_1_ was associated with changes in BMI, especially when consuming the HP/LGI diet. The TyG index is a good surrogate biomarker of IR [8–10, 59, 60], which is known to benefit from the type and quality of protein, as well as the glycemic index of the diet. These findings reveal that the TyG index is sensitive depending on the type of diet assigned, specifically the HP/LGI diet. In this context, these data are consistent with BMI being closely related to increased IR [1, 21], which is improved when following a high protein diet [48], since reductions in body fat are often associated with improved insulin sensitivity, which is now demonstrated with the TyG index. A recent study of a Chinese population (N = 116,661), demonstrated the causal association between TyG-BMI and DT2, focusing on the utility of this index, as it is simple, economical and reliable in medical practice to provide early detection and establish early preventive measures [15], being of great value in the field of personalized and precision medicine in primary clinical settings.

It is noteworthy that participants whose IR improved (the lower TyG index, the better insulin sensitivity) were found to have the most notable reductions in BMI, although it is also feasible that patients who lost more body weight had a concomitant more pronounced improvement of IR. This finding could be explained by the fact that not all patients with obesity have a metabolically healthy phenotype [61]. Interestingly, a subgroup of individuals in the population have a “metabolically healthy obesity phenotype” (approximately 30%), who despite having excess body fat, do not have comorbidities associated with obesity such as T2D, CVD and IR, among others, despite this term is under discussion [61, 62]. These patients might be expected to have a better response to therapeutic strategies than those presenting a “metabolically unhealthy obesity phenotype” [61], where genetic, transcriptional, and environmental factors associated with adipose tissue homeostasis and insulin sensitivity are involved [62]. Moreover, dietary weight loss intervention reduces insulin resistance, where mediation analyses revealed that decreased intrahepatic lipid content and insulin-induced muscle microvascular recruitment that independently contributed to improve insulin sensitivity, which also depends on body weight status [63].

Moreover, higher baseline weight predicts better management of body composition in patients with obesity [45]. Comparing the high and low glycemic index diets, no differences were found concerning TyG measurement, despite that some previous trials recognizing a beneficial role of fiber/low glycemic foods in managing IR, which confirms that the overall macronutrient composition, and not only carbohydrates, plays a metabolic role in IR management [64–67]. Indeed, LGI diets might be beneficial in patients with T2D [55, 57], since these patients with higher IR will benefit more from a low glycemic index diet due to a lower insulin demand to remove dietary carbohydrates present in the circulation [54], with increased impairment of pancreatic β-function and alteration of intestinal K-cell function [55, 57].

Dietary adherence is a major factor affecting weight changes in subjects with obesity, where metabolic flexibility also play a role [41, 68] as well as behavioral mechanisms [69]. In this context, different approaches have been devised for accounting caloric restriction including some based on energy expenditure measurement [70], despite the difficulties for energy imbalance quantification given homeostatic energy metabolism adaptations [41, 71] the assessment of body weight and composition has been used as a surrogate measure of energy balance in subjects with obesity [40]. Our results show that energy intake changes evaluated by body composition changes as a proxy may be a factor explaining the current weight loss outcomes as well as self-reported appetite and food preferences related to glycemic index and macronutrient distribution [52]. The changes in energy intake, protein consumption and glycemic index were compatible with the targeted values to demonstrate the hypothesis about the role of protein and glycemic index in weight loss maintenance after the planed dietary interventions to avoid weight regain.

The inter-individual differences concerning weight lowering interventions is recognized to depend on phenotypical traits and nutrigenetic/nutrigenomic interactions [72]. Previous data from the DIOGenes cohort have evidenced that initial fat stores may affect weight loss outcomes [7] and postprandial lipemia [73], as well as the metabolic adaptation [41]. Our results envisage that some individual differences in the response to diets could be attributed to different metabolic/obesity phenotypes in the current sample influenced by insulin resistance as has been reported concerning prediabetes development [74] or glycemic response considering integrative phenotyping, where the TyG index could contribute for precision prescriptions [36].

The strengths of the present research include the analysis of data belonging to the multicenter DIOGenes study with a considerable sample size, the use of standardized protocols for the collection of the clinical/anthropometric measurements, and the inclusion of potential confounding variables into the predictive models. However, some drawbacks in this investigation are related to the use of a *per protocol* analysis, leading to the loss of a number of patients, and thus, statistical power; the lack of some information concerning the diagnosis of chronic diseases and the use of therapies focused on lipid and glycemic managements; and the possible occurrence of type I and type II errors despite of statistical settings, could have influenced the results.

In conclusion, the current study showed that a HP/LGI diet is beneficial not only for weight maintenance after a LCD, but is also related to IR amelioration as assessed by TyG index changes. This knowledge may help to establish personalized nutrition/medicine strategies for obesity management and cardiometabolic improvement based on a single, economical and reliable surrogate for measuring IR with predictive application.

## Supplementary Information

**Additional file 1: Supplementary table 1.** TyG index correlation with BMI changes within complete nutritional program and weight maintenance stage.

## Data Availability

The data that support the findings of this study are available on request from the corresponding author [ORL].
