# Supplementary material for: Dietary protein and the glycemic index handle insulin resistance within a nutritional program for avoiding weight regain after energy-restricted induced weight loss
Source: Nutr Metab (Lond). 2022 Oct 19;19:71. doi: 10.1186/s12986-022-00707-y (PMC9583584; doi:10.1186/s12986-022-00707-y)
Supplement: Supplementary file 1 — Additional file 1: Supplementary table 1. TyG index correlation with BMI changes within complete nutritional program and weight maintenance stage. [file 12986_2022_707_MOESM1_ESM.docx]

| **Supplementary table 1** TyG index correlation with BMI changes within complete nutritional program and weight maintenance stage. | | | | |
| --- | --- | --- | --- | --- |
| **Change in BMI (kg/m^2^)**  †**Period 3 (0-34 weeks)** | ‡Types of diets | TyG_1_ | TyG_2_ | ∆ TyG_1_ |
|  | *Healthy diet (Control).* | 0.0710 | 0.0888 | 0.0006 |
|  | *LP/LGI* | -0.1520 | -0.0589 | -0.0936 |
|  | *LP/HGI* | 0.0145 | -0.0223 | 0.0297 |
|  | *HP/LGI* | 0.0157 | -0.2220* | 0.2110* |
|  | *HP/HGI* | 0.2684** | 0.1322 | 0.2109* |
| **Change in BMI (kg/m^2^)**  †**Period 2 (8-34 weeks)** | ‡Types of diets | TyG_1_ | TyG_2_ | ∆ TyG_1_ |
|  | *Healthy diet (Control).* | 0.1038 | 0.0312 | 0.0906 |
|  | *LP/LGI* | -0.0801 | -0.0523 | -0.0380 |
|  | *LP/HGI* | 0.0021 | -0.0552 | 0.0468 |
|  | *HP/LGI* | 0.0645 | -0.2560* | 0.2964 |
|  | *HP/HGI* | 0.3140* | 0.1487 | 0.2410* |
| Triglycerides-glucose index (TyG): TyG_1_ (basal value; pre-LCD), TyG_2_ (8 weeks; post-LCD) and the TyG changes for period 1 (ΔTyG_1_).  Correlation (r values and statistical direction).  *p-value of correlation test: *<0.05; **<0.001; ***<0.0001.  †*Period 3: Corresponds to the differences between baseline and final parameters encompassing the complete nutritional period (during 34 weeks); Period 2: Corresponds to the differences between the parameters after the low-calorie diet intervention (8 weeks) and after the nutritional treatment focused on weight maintenance for each type of randomized diet (8-34 weeks).*  ‡ *Types of diets: Control (healthy diet), LP/LGI (low protein, low glycemic index diet), LP/HGI (low protein, high glycemic index diet), HP/LGI (high protein, low glycemic index diet), HP/HGI (high protein, high glycemic index diet).* | | | | |
